# A Huge Asymptomatic Urachal Mucinous Cystic Carcinoma: A Case Report With Differential Diagnosis and Surgical Considerations

**DOI:** 10.7759/cureus.37036

**Published:** 2023-04-02

**Authors:** Adam Mylonakis, Lysandros Karydakis, Spyridon Davakis, Andreas Panagakis, Maria Kaniadaki, Alexandros Pergaris, Andreas Alexandrou, Alexandros Papalampros, Panagiotis Sakarellos

**Affiliations:** 1 First Department of Surgery, Laiko General Hospital, National and Kapodistrian University of Athens, Athens, GRC; 2 First Deparment of Pathology, Medical School, National and Kapodistrian University of Athens, Athens, GRC; 3 First Department of Pathology, Medical School, National and Kapodistrian University of Athens, Athens, GRC; 4 First Department of Surgey, Laiko General Hospital, National and Kapodistrian University of Athens, Athens, GRC

**Keywords:** urachus, mucinous, neoplasm, cystic neoplasm, urachal remnant

## Abstract

Cystic mucinous neoplasms of urachal origin cover a wide spectrum of benign and malignant lesions arising from the remnants of the urachus. They display various degrees of tumor cell atypia and local invasion, with no reported cases of metastasis or recurrence after complete surgical resection. We present a 47-year-old man who referred to our Surgical Department due to an abdominal cystic mass incidentally found upon abdominal ultrasound. He underwent en block resection of the cystic mass along with partial bladder dome cystectomy. The histopathology of the resected specimen revealed a cystic mucinous epithelial tumor of low malignant potential with areas of intraepithelial carcinoma. The patient showed no evidence of disease recurrence or distant metastasis 6 months after resection and is scheduled for follow-up with serial MRI or CT scans and blood tumor markers over the next 5 years.

## Introduction

The urachus is a tubular structure connecting the allantoid and the dome of the urinary bladder during fetal development, which is usually obliterated after birth and converted into the medial umbilical ligament. Occasionally in the event of incomplete atresia, urachal remnants consisting of tubular structures lined with transitional epithelium can be complicated with infection, cystic formation, or neoplasm development [1].

Urachal carcinoma is a rare clinical entity usually identified as a midline, supravesical mass, with mixed solid and cystic components that may be difficult to differential diagnose from other benign urachal lesions or urothelial bladder cancer [2]. It is an extremely rare malignancy, accounting for 0.01% of all cancers in adults [3], usually presenting with locally advanced disease [4-5].

Cystic mucinous neoplasms of urachal origin cover a wide spectrum of benign and malignant lesions from the common mucinous cystadenoma to the rare and aggressive frankly invasive mucinous cystadenocarcinoma. This category also includes mucinous cystic tumors of low malignant potential with/without intraepithelial carcinoma as well as mucinous cystadenocarcinoma with microinvasion [6].

In the present work, we report the case of a 47-year-old man who referred to our Surgical Department due to a cystic mucinous epithelial carcinoma of urachal origin and we mention our considerations on this rare tumor. 

## Case presentation

A 47-year-old man was referred to our Surgical Department for an abdominal mass. It was found incidentally upon abdominal ultrasound after being admitted to another hospital with symptoms of fatigue and palpitations. The patient's medical history revealed a Hodgkin Lymphoma in remission after treatment 30 years ago, hypertension, dyslipidemia, complete atrioventricular block with a dual-chamber rate-modulated (DDDR) pacemaker implantation. He had a history of tobacco use, having smoked for a period equivalent to 25 pack years. The physical examination of chest and abdomen was unremarkable. A chest X-ray was normal. Complete blood count and biochemical tests were within normal range. On admission, the patient exhibited slightly elevated serum level of carcinoembryonic antigen (CEA) of 6.12 ng/mL (normal range <5.0 ng/mL) and elevated serum level of carbohydrate antigen 19-9 (CA 19-9) of 72.50 U/mL (normal range <34 U/mL). Colonoscopy was unremarkable other than a microscopic polyp which was endoscopically removed. The patient underwent a hematological assessment due to the past medical history of Hodgkin Lymphoma, which was negative for disease recurrence.

Ultrasound examination of the abdomen revealed a lower abdomen mixed echo mass with cystic and solid segments separated by septa. The lesion measuring 224 mm x 141 mm displayed a blood flow towards the more solid areas of the mass.

**Figure 1:**
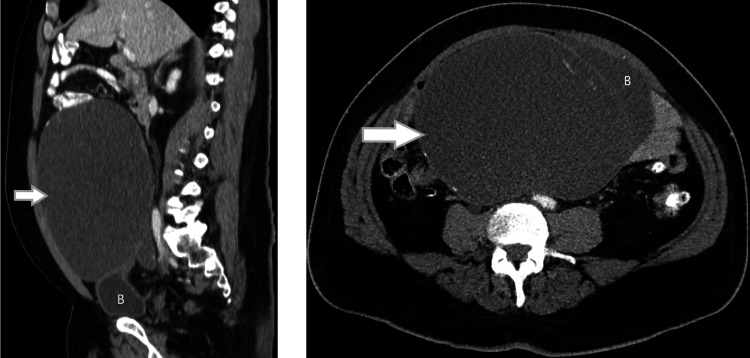
Sagittal and axial image from contrast enhanced CT demonstrating a cystic tumor (see arrow) arising from the dome of the urinary bladder (labeled “B”) corresponding to a urachal cystic tumor of low malignant potential.

A contrast-enhanced CT scan of the abdomen (Figure 1) demonstrated a cystic lesion of liquid consistency measuring 241 mm x 145 mm x 240 mm, extending from the level of the transverse colon into the lesser pelvis. The lesion showed no enhancement after contrast infusion and had internal septa and calcifications of its wall. The mass displaced the adjacent small intestine without invading it, pressured the middle part of right ureter, causing mild distension of the right ureter and of the right renal pelvis and was associated with the superior apex of the urinary bladder. Due to the extended anatomical continuity of the mass with the upper pole of the urinary bladder, a diagnosis of urachal remnant origin was put forth, as well as of lesion of mesenteric origin.

**Figure 2:**
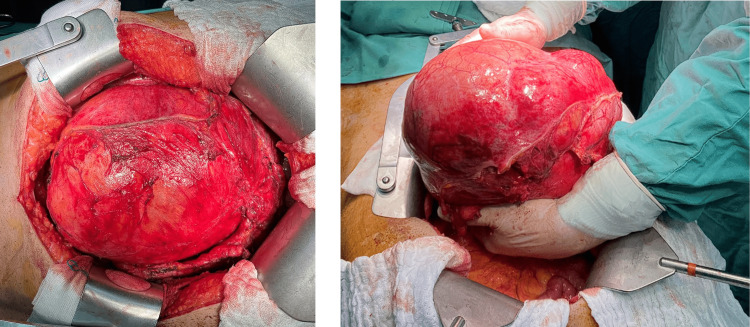
Intraoperative image of the urachal cystic mass excised en bloc with the dome of the urinary bladder.

After multidisciplinary team (MDT) council, surgical excision of the tumor was scheduled. After bilateral ureteral pigtail placement for ureter protection, we performed an en block resection of the cystic mass along with partial bladder dome cystectomy (Figure 2), while confirming the absence of leakage from the urinary bladder. Intraoperatively, we did not observe signs of pseudomyxoma peritonei. Postoperatively, the patient made an uneventful recovery and was discharged on the seventh postoperative day.

**Figure 3:**
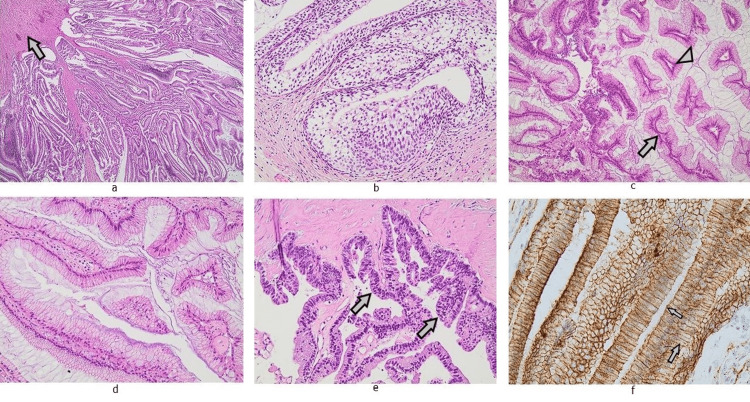
Microscopic appearance of the cystic mass: on low power, the papillary architecture of the epithelium can be appreciated over a thick muscular layer (arrow). (a) (H/E, x20) Non-neoplastic urachal remnants were identified adjacent to the mass. (b) (H/E, x200) Epithelium comprised columnar cells with intracellular mucin lining fibrovascular cores (arrow), forming true papillae that protruded into pools of acellular mucin (arrowhead). (c) (H/E, x100) Cytologic atypia was predominantly mild. (d) (H/E, x200) Foci of high cellular atypia were rarely seen (arrow). (e) (H/E, x200) On immunohistochemistry, neoplastic cells showed positive cytoplasmic beta catenin staining with membrane attenuation (arrows). (f) (beta catenin stain, x400).

The resected cystic mass measured 230 mm x 220 mm x 190 mm with a wall thickness of 1-19 mm and was filled with a cream-colored, gelatinous fluid. Histologic examination of the specimen revealed a cystic lesion with well-developed, thick smooth muscle wall lined by columnar, mucin-producing epithelium with papillary architecture (Figure 3). Cytologic atypia was predominantly mild with rare foci of high-grade dysplasia. The elongated, glandular formations projected into pools of acellular mucin with no evidence of invasive growth. On immunohistochemistry, neoplastic cells exhibited strong positivity for CDX2 and CK20 along with weak expression of cytokeratin 34 beta E12. No CK7, p63, GATA-3, and SATB2 expression was encountered. p53 immunostaining showed a wildtype pattern of expression and beta catenin cytoplasmic expression with membrane attenuation.

The normal p53 expression pattern, weak cytokeratin 34 beta E12 expression along with the absence of nuclear beta catenin staining and the lack of association with colon tissues, as confirmed by a number of imaging studies carried out that included a colonoscopy and a CT scan, ruled out colon adenocarcinoma. The emergence of the lesion from the dome of the urinary bladder along with its morphological and immunohistochemical characteristics was consistent with an urachal mucinous cystic tumor of low malignant potential (MCTLMP) with areas of intraepithelial carcinoma [7-14].

The patient did not receive any adjuvant treatment and six months after resection was followed-up with CT series scan that showed no evidence of disease recurrence or distant metastases. He is scheduled for a close surveillance with MRI or CT series scan and CEA, CA19-9 serum level tests over the next 5 years.

## Discussion

The urachus is a tubular structure of allantoic origin which obliterates after birth forming the medial umbilical ligament. Microscopic remains of the urachus are identifiable in most adult, and in one third of these, the remnants remain in contiguity with the urinary bladder lumen [15]. Neoplasms may arise from the remnant epithelium of the urachus and mainly consist of adenocarcinoma (90%) [16]. They are believed to have evolved from intestinal metaplasia of the transitional epithelium [6].

Mucinous neoplasms of the urachus represent the majority of the urachal neoplasms, accounting for 31 of the 55 glandular neoplasm of the urachus based on the largest cohort presented in the literature [6]. Due to their homology, mucinous neoplasms of the urachus are subclassified in accordance with the scheme used for the mucinous surface epithelial tumors of the ovary based on the epithelial proliferation, cellular atypia, and stromal invasion. Lesions are characterized as mucinous cystadenoma, cystic tumor of low malignant potential with/without intraepithelial carcinoma, cystadenocarcinoma with microinvasion, and cystadenocarcinoma with frank invasion [6]. To our knowledge, our case is the 48th case of mucinous urachal neoplasm and the 26th case of mucinous cystic tumor of low malignant potential.

Urachal tumors usually remain latent for long period because of their obscured location between the dome of the bladder and the umbilicus. They mostly present as an abdominal mass (32% of cases) or incidental finding (32% of cases) as well as with hematuria, mucusuria, or pain [6]. Similar to other adenocarcinomas of enteric type, carcinomas arising from the urachus can present with elevated levels of CEA and carbohydrate antigen 19-9 (CA 19-9)[4].

The differential diagnosis of urachal neoplasms includes a wide range of benign and malignant conditions. The most common urachal malignancy is urachal adenocarcinoma, but other primary malignancies of the urachus are also possible, including sarcoma, lymphoma, and small cell carcinoma. Metastatic tumors from other sites can also involve the urachus. Benign urachal masses such as cysts, diverticula, and adenomas can present similarly to malignant tumors. Inflammatory and infectious conditions, such as urachitis, tuberculosis, and fungal infections, should also be considered in the differential diagnosis.

Radiologic findings reveal a midline mass anterosuperior to the dome of the bladder that can be solid, cystic, or mixed. CT and MRI series provide further information on disease extension, regional lymph node involvement and the presence of distant metastases [17]. Especially mucinous carcinomas may produce calcifications and their presence is considered nearly pathognomonic for adenocarcinoma [17].

Considering the spectrum of atypia within the same cystic mass, sampling can be a hazard of pathologic evaluation. Proper and extensive biopsy should be performed if atypia is found on initial assessment, or in cases of a multilocular lesion, so as to identify potentially small areas of carcinoma that have potential prognostic value [6, 12].

As far as morphological features are concerned, mucinous urachal neoplasms are mainly unilocular, filled with mucin exhibiting often areas of calcification and rarely ossification [6]. Tumor cell atypia and mitosis, growth pattern, and invasion are the main characteristics for mucinous cystic neoplasmatic subclassification.

The treatment of choice for patients with mucinous cystic neoplasms of the urachus is mass surgical excision, with possible urachectomy or partial cystectomy. The largest series of this type of tumor in the current literature of 39 cases, including three patients with intraepithelial and invasive carcinoma, exhibited no recurrence or metastasis on follow-up after surgical resection. Thus, although the data remain limited, these patients appear to have a favorable prognosis after complete excision [6].

In our study, we present the case of a 47-year-old man who was referred to our department due to an abdominal mass found incidentally upon abdominal ultrasound. Diagnostic workup included blood and liver function tests, cancer markers, and CT of the abdomen that raised the suspicion of a cystic mucinous neoplasm arising from the urachus. We performed an en block resection of the cystic mass along with partial bladder dome cystectomy. The resected mass measured 24 cm at its greatest diameter; the second largest reported in the literature, following the one described by Neo and Lee [18]. 

Taking into consideration the location, the morphological characteristics and the immunohistochemical profile of the resected tumor, the initial differential diagnosis included a villous urachal adenoma of lower urinary tract, a urothelial carcinoma with glandular differentiation, a primary adenocarcinoma of the bladder, a secondary colorectal adenocarcinoma of colloid type as well as mucinous urachal tumors and mucinous tumors of the appendix.

Villous urachal adenomas arise in the dome of the bladder or in the urachus and often comprise a noninvasive, superficial component of an adenocarcinoma. Histologically, they show elongated, finger-like projections with central fibrovascular cores lined by pseudostratified columnar epithelium with variable amounts of intracytoplasmic mucin. Extracellular mucin may also be present. In contrast with the neoplasm of our case, these lesions are grossly polypoid and non-cystic. Moreover, these adenomas are described as forming an excrescent focus of dysplastic mucinous epithelium arising in the setting of otherwise nondysplastic metaplastic urachal remnant epithelium tissues and do not present as mucinous cystic urachal neoplasms with multiple foci of atypia along their lining, as the one in our case [7].

Invasive urothelial carcinoma of the bladder with glandular differentiation and primary adenocarcinoma of the bladder-especially the mucinous type-needed to be excluded as well. The distant location of the neoplasm from the bladder’s epithelium as well as the sharp delineation between the tumor’s and bladder’s epithelium by a thick muscular layer along with the normal appearance of the urinary bladder, as observed through both imaging studies and intraoperatively, ruled out a primary bladder neoplasm.

Colorectal adenocarcinoma of colloid variant has almost similar morphological and immunophenotypic characteristics with urachal mucinous tumors. However, the immunohistochemical findings described above (lack of diffuse nuclear b-catenin staining, lack of expression of CK7 along with positive keratin 34βE12 staining), the absence of atypical free cells floating in extracellular pools of mucin in correlation with normal colonoscopic and imaging (CT scan) findings excluded the colonic origin of the neoplasm.

The mucinous epithelium of the tumor presented has also many similarities with mucinous neoplasms of appendiceal origin, especially with a low grade mucinous appendix neoplasm (LAMN). These neoplasms consist of mucinous epithelium with low grade atypia and have the potential to spread through the visceral peritoneal surface of the appendix into the peritoneal cavity -and thus in the bladder- forming mucinous, acellular deposits. This condition is called “pseudomyxoma peritonei” and is clinically recognizable. However, the patient’s appendix and peritoneal cavity appeared normal both intraoperatively and radiologically.

Urachal adenocarcinomas are glandular neoplasms with specific diagnostic criteria as modified by Johnson et al. [19]. These include the location of the tumor in the dome of the bladder or anterior wall, epicenter of the tumor in the bladder wall, absence of widespread cystitis glandularis or intestinal metaplasia beyond the dome or anterior wall as well as absence of urinary neoplasia and other primary tumor elsewhere. The coexistence of urachal remnants is a leading but not definitive criterion for diagnosis of urachal neoplasms [7]. The mucinous cystic tumor of our case was located in the dome of the bladder, in its deeper muscularis propria tissues, with a sharp demarcation area from the surface of urinary bladder mucosa. Urachal remnants were also detected in adjacent positions. The epithelium of the cyst was glandular and dysplastic but no invasive component in the muscular layer was observed. Any other origin in the differential diagnosis has been previously ruled out. Taking into consideration all the aforementioned criteria, a diagnosis of a urachal mucinous cystic tumor of low malignant potential (MCTLMP) with areas of intraepithelial carcinoma was reached.

Considering the rarity of these tumors as well as the potential involvement of adjacent structures, we suggest that all case of suspected or diagnosed urachal neoplasms be referred to tertiary hospitals with specialized surgical and urological units. We note the importance of en bloc resection and proper surgical planning in order to ensure negative resection margins and to avoid tumor tear and spillage. We highlight the importance of an MDT approach composing of an oncologist, surgeon, urologist, diagnostic and interventional radiologist, and pathologist to ensure optimal patient outcomes.

At present, more data and case series are required to establish therapeutic guidelines for mucinous cystic neoplasms of urachus. Although the prognosis of these patients appears to be favorable based on current evidence, we suggest close and long-term follow-up after complete surgical resection.

## Conclusions

Mucinous cystic neoplasms of urachal origin cover a wide spectrum of benign and malignant lesions that based on current data published, appears to have a favorable prognosis after complete surgical resection. More case series with close patient follow-up are required to establish therapeutic and follow-up guidelines.
